# The Psychological Benefits of Breastfeeding: Fostering Maternal Well-Being and Child Development

**DOI:** 10.7759/cureus.46730

**Published:** 2023-10-09

**Authors:** Anushree Modak, Vaishnavi Ronghe, Kavita P Gomase

**Affiliations:** 1 Obstetrics and Gynaecology, Smt. Radhikabai Meghe Memorial College of Nursing, Datta Meghe Institute of Higher Education & Research, Wardha, IND; 2 Obstetrics and Gynaecological Nursing, Smt. Radhikabai Meghe Memorial College of Nursing, Datta Meghe Institute of Higher Education & Research, Wardha, IND

**Keywords:** awareness, oxytocin, child development, maternal well-being, psychological benefits, breastfeeding

## Abstract

The value of breastfeeding surpasses its utilitarian role in nourishing, encompassing profound psychological advantages for mothers and children. The orchestration of emotional bonds relies on the interplay of oxytocin and prolactin, fundamental hormones that underpin maternal attachment, mitigate postpartum depression, and cultivate self-confidence. Simultaneously, breastfeeding promotes infant development by fostering robust brain growth, bolstering immune defenses, and nurturing cognitive and emotional maturation - all of which are nurtured through maternal interactions. We must respond to the call for heightened advocacy of breastfeeding. This entails delivering education, easily accessible support, and creating an environment where breastfeeding is normalized. By dispelling misconceptions and eradicating stigmatization associated with breastfeeding, we can amplify awareness and empower mothers to make well-informed decisions for their newborns. These implications reverberate extensively. Enhanced maternal mental well-being and self-assurance form the bedrock of healthier family dynamics. At the same time, the dividends of cognitive, emotional, and immunological enrichment in children represent a more promising future. At a societal level, the embrace and promotion of breastfeeding cultivate an environment that places immense value on the health and happiness of both mothers and children. This journey is more profound than mere sustenance; it signifies a complex web of advantages. Elevating awareness and support for breastfeeding solidifies the global commitment to comprehensive maternal and child welfare and the flourishing of meaningful relationships.

## Introduction and background

Breastfeeding plays a pivotal role in ensuring the health and well-being of mothers and children. Beyond its extensively documented nutritional advantages, breastfeeding is central to establishing the foundation of robust maternal and child health. The act of breastfeeding not only supplies essential nutrients but also nurtures a profound emotional and psychological connection between the mother and her infant. Therefore, understanding the broader implications of breastfeeding for maternal well-being and child development is crucial in advancing comprehensive healthcare strategies [1,2].

While the physical merits of breastfeeding are firmly established, there has been a growing focus in recent years on its psychological dimensions. This comprehensive review delves deeply into the psychological facets of breastfeeding and how this intimate and nurturing practice significantly contributes to the emotional health and development of both mothers and their children. By exploring the intricate interplay between hormones, neural pathways, and emotional bonds, we aim to illuminate the substantial and multifaceted benefits beyond breastfeeding's nutritional content [3].

This review article's primary aim is to thoroughly examine the psychological advantages linked to breastfeeding, encompassing maternal well-being and child development. By synthesizing current research, we strive to spotlight the intricate mechanisms that underlie the positive influence of breastfeeding on maternal mental health, the cultivation of bonding and attachment, and its contribution to optimal child development. Moreover, we seek to underscore the importance of supportive environments, societal attitudes, and healthcare policies in fostering breastfeeding as a holistic approach that enhances the overall health and happiness of mothers and their children.

## Review

Psychological benefits of breastfeeding for mothers

Release of Oxytocin and Bonding With the Baby

The release of oxytocin, often called the "bonding hormone," represents one of the most profound psychological benefits of breastfeeding. Oxytocin is a neurochemical released during breastfeeding and skin-to-skin contact, playing a pivotal role in fostering a deep emotional connection between the mother and her baby. This hormone is instrumental in promoting love, trust, and attachment, forming the cornerstone of a secure and nurturing relationship between mother and child. As mothers breastfeed, they engage in moments of close physical proximity and intimacy, contributing to the establishment of a robust maternal-infant bond. Research indicates that oxytocin's influence on bonding enhances the mother's and child's overall emotional well-being [4].

Reduction of Postpartum Depression and Anxiety

Breastfeeding's positive impact on maternal mental health is noteworthy, with a demonstrated reduction in the risk of postpartum depression and anxiety. The release of oxytocin during breastfeeding strengthens the maternal-infant bond and aids in regulating stress and mood. Breastfeeding promotes focused attention and relaxation, akin to a meditative experience, which can effectively alleviate sadness and anxiety. Additionally, the structured routine established through breastfeeding can contribute to a sense of predictability and stability, essential components in mitigating postpartum emotional challenges [5].

Increased Self-Esteem and Maternal Confidence

Breastfeeding often leads to an increase in self-esteem and maternal confidence. Directly providing nourishment and sustenance from a mother's body generates a profound sense of accomplishment and capability. Conquering the obstacles associated with breastfeeding, such as perfecting latch techniques and ensuring an adequate milk supply, cultivates a newfound sense of mastery and self-assuredness. Significantly, this elevated confidence level extends beyond breastfeeding, positively influencing mothers' perceptions of their parenting capabilities [6].

Sense of Accomplishment and Empowerment

Breastfeeding is a fundamental and intrinsic facet of motherhood, giving women a sense of accomplishment and empowerment as they nourish their infants in this distinct manner. The ability to sustain and nurture a growing baby through their bodies reinforces a profound connection to the elemental roles of motherhood across human history. This shared human experience fosters a sense of purpose and empowerment, equipping mothers with the ability to navigate the challenges of early parenthood while experiencing a heightened sense of fulfillment [2].

Child developmental benefits of breastfeeding

Nutritional Advantages for Optimal Brain Development

Breast milk as a reservoir of essential nutrients: breast milk is often called "liquid gold" due to its incredible composition of essential nutrients tailored to meet an infant's growth and development needs. It contains many vitamins, minerals, proteins, fats, and carbohydrates that support the baby's overall health, significantly contributing to brain development [2].

Docosahexaenoic acid (DHA) and arachidonic acid (ARA): two crucial components found in breast milk are DHA and ARA. These are omega-3 and omega-6 fatty acids, respectively, that play a pivotal role in cognitive growth and visual acuity. DHA, in particular, is a major structural component of the brain and the retina of the eyes. It is crucial for the development and maintenance of neural pathways, synapses, and cell membranes in the brain, thereby influencing learning, memory, and overall cognitive function. ARA also contributes to brain and nervous system development and supports various cellular processes [2].

Building blocks for brain tissue: both DHA and ARA act as building blocks for brain tissue. During the rapid growth phase of infancy and early childhood, the brain undergoes significant structural and functional development. These fatty acids form new neural connections and intricate pathways that enable the brain to process information, make decisions, and engage in problem-solving.

Cognitive growth and visual acuity: cognitive growth refers to the development of various cognitive abilities, including language, attention, memory, and problem-solving skills. Visual acuity, on the other hand, relates to the sharpness and clarity of vision. The presence of DHA and ARA in breast milk helps ensure that the infant's brain and visual system develop optimally, setting the stage for a child's ability to perceive the world, understand it, and interact with it effectively [3-4].

Support for learning and problem-solving: the neural pathways established during early brain development lay the foundation for a child's capacity to learn, retain information, and engage in effective problem-solving throughout life. Adequate intake of DHA and ARA during infancy through breastfeeding can contribute to enhanced cognitive abilities, which can have a lasting impact on a child's academic performance, social interactions, and overall success [5].

Robust nutritional bedrock: breast milk's unique composition provides infants with a robust nutritional foundation that supports their growth and development during the critical early years of life. While formula milk attempts to replicate some of these nutritional components, breast milk remains the gold standard due to its tailored and dynamic nature that adapts to the growing baby's changing needs [6-7].

Role of Breast Milk in Building a Strong Immune System

Breast milk is a remarkable substance and its benefits go beyond mere nutrition. It is a complex fluid that provides many immunological benefits crucial for developing a strong immune system in infants. This is due to breast milk's multifaceted immune-boosting attributes, which actively contribute to fortifying the infant's immune system. A newborn's immune system is not fully developed, making them particularly susceptible to various infections and illnesses. However, nature has equipped mothers with a powerful tool to help their infants combat these challenges: breast milk. Breast milk is a dynamic source of passive immunity, transferring various immunological factors from mother to child. These factors are crucial in bolstering the infant's immunity during their initial months of life [6].

One of the key components transmitted through breast milk is antibodies. Antibodies are proteins the mother's immune system produces in response to infections or vaccinations. When these antibodies are passed to the infant through breast milk, they offer protection against various pathogens. This early protection can be vital in preventing severe infections and reducing the severity of illnesses. White blood cells, another essential immune system element, are also present in breast milk. These cells are fundamental in detecting and fighting off harmful microorganisms. By providing these cells through breast milk, mothers are effectively arming their infants with an added layer of defense against potential threats [7].

Beyond antibodies and white blood cells, breast milk contains a diverse array of bioactive compounds. These compounds include cytokines, enzymes, growth factors, and other molecules in immune modulation, inflammation regulation, and tissue development. Collectively, these compounds contribute to the overall immune-boosting properties of breast milk. The process of breastfeeding itself offers more than just immunological benefits. Skin-to-skin contact during breastfeeding, for instance, promotes the transfer of beneficial bacteria from the mother to the infant, thereby shaping the infant's developing gut microbiota. A balanced gut microbiome is increasingly recognized as critical in immune system development and overall health. The culmination of all these immune-boosting attributes results in breastfed infants having a significant advantage in immune protection. Breast milk not only helps prevent infections but also contributes to the overall health and well-being of the infant. Studies have shown that breastfed infants experience fewer respiratory infections, gastrointestinal illnesses, and allergies than formula-fed infants [8].

Cognitive and Emotional Development Through Maternal Interaction

The cognitive and emotional development process in infants is a complex and fascinating journey, heavily influenced by the interactions between mothers and their children. One particularly significant aspect of this development is the act of breastfeeding, which serves as a dynamic platform for nurturing cognitive and emotional growth. Breastfeeding is not just about providing essential nutrition; it is a multifaceted experience beyond transferring nutrients. It is a time when the mother and child come together uniquely and intimately. The quoted text beautifully describes this experience as a "symphony" orchestrated by various elements [3].

Physical closeness: breastfeeding creates a close physical bond between the mother and the infant. The infant feels the warmth and comfort of the mother's body, enhancing feelings of security and closeness. This physical proximity triggers a cascade of physiological responses contributing to emotional well-being [4].

Direct eye contact: mothers and infants often use direct eye contact during breastfeeding. This visual exchange is crucial not only for ensuring proper latching but also for promoting emotional connection. Eye contact is a powerful means of conveying affection, love, and attention. This interaction lays the foundation for developing trust and emotional reciprocity [5].

Skin-to-skin touch: the gentle skin-to-skin touch that accompanies breastfeeding has numerous benefits. It regulates the infant's body temperature, heart rate, and breathing. But beyond these physiological effects, touch is a primal form of communication. It fosters a sense of security, soothing the infant and creating positive associations with feeding [6].

All these elements of maternal interaction during breastfeeding trigger the release of hormones such as oxytocin and prolactin. Oxytocin, often called the "love hormone," enhances social bonding, trust, and emotional attachment. Prolactin supports milk production and is associated with feelings of nurturing and maternal care. These hormones, acting as "architects," as described in the text, are crucial in shaping the infant's brain development and emotional balance [7].

Long-Term Effects on Reducing the Risk of Chronic Diseases

Nutritional content: breast milk is uniquely tailored to meet an infant's nutritional needs. It contains a balanced combination of essential nutrients, vitamins, and minerals that contribute to the healthy growth and development of the child. These early nutritional experiences could potentially influence metabolic processes and set the foundation for better health in the long term [8].

Immunological factors: breast milk contains many immunological components such as antibodies, white blood cells, and enzymes that help bolster the infant's immune system. This enhanced immune response during infancy might improve immune function, potentially reducing the risk of chronic inflammatory conditions linked to diseases like obesity and cardiovascular issues [8].

Metabolic programming: early life experiences, including nutrition during infancy, can profoundly impact metabolic programming. Breastfeeding might influence gene expression, hormonal regulation, and metabolic pathways, affecting how the body processes energy and manages metabolic functions later in life. This programming could play a role in reducing the risk of obesity and related conditions [8-9].

Microbiota and gut health: breast milk contributes to the establishment of a healthy gut microbiota in infants. A balanced gut microbiome is increasingly recognized as crucial for overall health, including metabolic and immune function. Through breastfeeding, positive effects on gut health might contribute to long-term health benefits [9].

Bonding and attachment

Skin-to-Skin Contact and Its Impact on Mother-Infant Attachment

Skin-to-skin contact holds remarkable significance in shaping the attachment between mothers and infants. This intimate practice, often intertwined with breastfeeding, wields the capacity to initiate a profound emotional bond. Through skin-to-skin moments, the physical closeness triggers the release of oxytocin, commonly known as the "bonding hormone." This biological response elicits comfort, security, and a profound connection. A critical outcome of such interactions is the amplification of the mother's receptiveness to her baby's cues and requirements. The physical closeness and direct skin contact facilitate a heightened awareness of the infant's nonverbal signals, fostering an intuitive understanding of their needs. This sensitized responsiveness becomes pivotal in building trust and emotional intimacy as the mother learns to promptly interpret and meet her baby's needs [10].

Beyond its emotional dimensions, skin-to-skin contact assumes a practical role in facilitating successful breastfeeding. The proximity during these moments can trigger the baby's natural feeding reflexes, often leading to a smoother latch and more effective breastfeeding sessions. This supports the baby's nutritional needs and bolsters the emotional bond between mother and child. The enduring impact of skin-to-skin contact is the establishment of a robust and enduring mother-infant bond. The foundation of trust and intimacy cultivated through these interactions is a cornerstone for secure attachment. Research has shown that skin-to-skin practices in the early postpartum can contribute to more positive developmental outcomes, including enhanced cognitive and emotional growth [10].

Development of Secure Attachment and Emotional Regulation

Secure attachment: secure attachment refers to the emotional connection and bond between an infant and their primary caregiver, usually the mother. This attachment is characterized by the child feeling safe, loved, and confident in the caregiver's presence. Securely attached infants actively explore their environment and develop healthy social and emotional skills. They have a foundation of trust that allows them to seek comfort and support from their caregiver when needed. Breastfeeding contributes to the development of secure attachment through consistent and responsive interactions between the caregiver and the infant. When a mother breastfeeds, she holds the baby close, providing physical closeness, eye contact, and skin-to-skin contact. These interactions foster a sense of intimacy and connection between the caregiver and the infant, helping the infant build a strong emotional bond [9]. The act of breastfeeding also involves the release of hormones like oxytocin, often referred to as the "love hormone." Oxytocin promotes feelings of warmth and connection between the mother and the baby. These positive interactions and emotional experiences create a foundation for the infant's understanding of relationships, forming the basis for future social interactions [10].

Emotional regulation: emotional regulation refers to managing and controlling one's emotions in response to different situations. It is an essential skill for overall emotional well-being and healthy social interactions. Infants are born with limited emotional regulation abilities and rely heavily on their caregivers to help them regulate their emotions [10]. Breastfeeding plays a role in developing emotional regulation skills by providing a source of comfort and soothing during times of distress. When a baby is breastfed, they experience physical closeness, warmth, and nourishment, which can help reduce stress and anxiety. Sucking during breastfeeding also has a calming effect on the baby's nervous system. Through consistent breastfeeding interactions, infants learn that their caregiver is a reliable source of comfort and support. This understanding fosters a sense of security, which allows the infant to develop the ability to self-soothe and regulate their emotions gradually. Over time, the infant begins to internalize these experiences and strategies, contributing to their growing emotional resilience and self-regulation skills [11].

Role of Breastfeeding in Promoting Responsive Parenting

The role of breastfeeding in promoting responsive parenting is multifaceted and significant. Breastfeeding goes beyond being a means of providing essential nutrition; it plays a crucial role in fostering a nurturing and attentive caregiving approach that benefits both the mother and the infant. This process involves a deep connection between the physical act of breastfeeding, the emotional bonding it facilitates, and establishing a responsive parenting style [9].

Recognition of infant's needs: breastfeeding requires frequent and intimate interactions between the mother and the infant. Nursing encourages the mother to be consistently present and attuned to her baby's cues. As she spends time feeding and nourishing her baby, she becomes more adept at recognizing subtle signs and signals that indicate the infant's needs - hunger, discomfort, or a need for comfort. This heightened awareness forms the basis of responsive parenting, where the mother is better equipped to promptly address her baby's needs [10].

Enhanced bonding and attachment: the physical closeness that breastfeeding entails, including skin-to-skin contact, triggers the release of oxytocin - often referred to as the "love hormone" or "bonding hormone." Oxytocin fosters emotional connection, trust, and attachment between the mother and the infant. This bond is crucial for developing a secure and healthy attachment-based relationship. This secure attachment is a foundation for the child's emotional and psychological development, influencing their future relationships and well-being [11].

Modeling effective communication: breastfeeding establishes a communication dynamic between the mother and the infant. During nursing sessions, the baby's cues are met with the mother's timely responses - whether through feeding, soothing, or comforting. This interaction models a form of communication in which the baby's nonverbal cues are acknowledged and appropriately addressed. This experience of being understood and responded to lays the groundwork for effective communication and empathy in the child's later interactions [12].

Emotional regulation: responsive parenting nurtures emotional regulation in infants. When a mother consistently responds to her baby's needs, the infant learns that their emotions and distress will be addressed. This sense of security and predictability fosters emotional regulation, enabling the child to learn how to manage their emotions healthily. This skill is vital for their well-being and social development [13].

Nurturing a caregiving approach: breastfeeding mothers often adopt a caregiving approach characterized by sensitivity, warmth, and attentiveness. The act of nurturing through breastfeeding extends to other aspects of caregiving, where mothers are more likely to respond to their baby's signals promptly and empathetically. This approach creates a nurturing environment where the baby feels valued and understood [14].

Hormonal and neurological influences

Oxytocin's Role in Fostering Maternal Caregiving and Bonding

Physiological aspects: oxytocin is primarily known for stimulating uterine contractions during labor and promoting the milk ejection reflex during breastfeeding. These physiological effects are crucial for childbirth and maternal care. When a baby suckles at the mother's breast, sensory receptors in the nipple are stimulated, releasing oxytocin from the brain's hypothalamus. This release causes the milk to flow through the ducts, allowing the baby to feed [14].

Emotional aspects: beyond its physiological effects, oxytocin has profound emotional implications as well. It has been dubbed the "love hormone" or "bonding hormone" because of its involvement in social and emotional behaviors. Oxytocin is released during positive social interactions, such as hugging, touching, and intimate moments. It plays a role in creating feelings of attachment, trust, and emotional closeness. In maternal caregiving, oxytocin fosters a strong emotional bond between the mother and her baby [14].

Maternal caregiving and bonding: oxytocin's influence on maternal caregiving is remarkable. When oxytocin is released during breastfeeding, it not only aids in milk ejection but also has a calming and soothing effect on the mother. This can create a positive feedback loop: the mother's relaxation releases more oxytocin, reinforcing her positive emotional state. This cycle can significantly contribute to the mother's feelings of warmth and affection toward her baby [14].

Attachment and trust: oxytocin is closely tied to developing a secure attachment between a mother and her baby. Attachment refers to the emotional bond that forms between an infant and their primary caregiver. Oxytocin encourages the mother to respond sensitively to her baby's cues and needs. When the mother consistently meets the baby's needs and responds with affection and care, the baby develops a sense of security and trust. This lays the foundation for healthy emotional and social development [14].

Empathy and emotional regulation: oxytocin also promotes empathy, which is essential for understanding and responding to the emotions of others. This is crucial in caregiving, as it helps the mother attune to her baby's emotional state. Additionally, oxytocin is linked to emotional regulation, helping the mother and the baby manage stress and anxiety. This regulation is vital for maintaining a nurturing and secure environment for the baby's growth [14].

Prolactin and Its Effects on Maternal Behavior and Nurturing Instincts

Prolactin is a hormone produced by the pituitary gland, a small gland located at the base of the brain. Its primary role is to stimulate and regulate milk production in the mammary glands of mammals, including humans. During pregnancy, prolactin levels rise, preparing the breasts for milk production. After childbirth, when a mother starts breastfeeding, prolactin levels experience a surge. This surge in prolactin is essential for initiating and maintaining lactation - the process of producing and releasing milk to nourish the newborn [15].

Broader effects on maternal behavior: while prolactin's primary function is related to milk production, it also has a broader range of effects on maternal behavior and emotions. This hormone has been found to influence various aspects of a mother's behavior and psychology, contributing to her ability to nurture and care for her baby. Some of the notable effects of prolactin are described below.

Elevated prolactin levels during breastfeeding encompass a range of effects that profoundly influence maternal behavior and nurturing instincts. These effects extend beyond the hormone's primary role in milk production, encompassing emotional and behavioral dimensions that enhance the mother-child relationship. Mothers experience a dual benefit of relaxation and contentment due to increased prolactin levels. This tranquil state creates an ideal setting for bonding and caregiving as mothers become more attuned to their baby's cues and needs, establishing a strong emotional connection [15].

Simultaneously, prolactin enhances a mother's focus on caregiving tasks. This heightened attentiveness enables swift responses to the baby's demands - from feeding to providing comfort. The augmented dedication to these tasks fosters a nurturing environment vital for the infant's holistic development. Prolactin's role in activating and amplifying nurturing instincts is crucial. These instincts drive behaviors that promote bonding, including a heightened preference for physical closeness. Holding, cuddling, and skin-to-skin contact become exceptionally rewarding and essential for both mother and baby under the influence of prolactin. This hormone also cultivates more attentive interactions. Mothers with elevated prolactin levels exhibit a remarkable sensitivity to their baby's cues, expressions, and vocalizations, allowing them to deliver well-timed and appropriate care [15].

Impact of Breastfeeding on Stress Reduction for Both Mother and Child

Breastfeeding yields a tranquilizing influence that benefits both the mother and the child, leading to stress reduction through a range of mechanisms. In the mother's case, breastfeeding initiates the release of oxytocin, a hormone that counteracts the impact of stress hormones such as cortisol. This hormonal reaction fosters a state of relaxation and overall well-being for the mother. Conversely, breastfeeding serves as a source of solace and pacification for the infant through skin-to-skin contact, rhythmic suckling, and the sensory experience of breast milk's taste and aroma. These pacifying effects are crucial in regulating the baby's stress response and cultivating a sense of security and ease [16].

Psychological well-being of mothers

Positive Effects on Maternal Mental Health and Overall Happiness

Breastfeeding has many beneficial impacts on maternal mental health and overall well-being. The secretion of hormones such as oxytocin and prolactin during breastfeeding plays a pivotal role in inducing sensations of calmness, joy, and emotional harmony. These hormones act as natural stress mitigators and facilitators of a positive emotional state. Furthermore, breastfeeding cultivates a sense of accomplishment and contentment, elevating a mother's self-regard and contentment with her role as a nurturer. Breastfeeding engenders a profound sense of fulfillment, bolstering the mother's self-esteem and fostering a deep satisfaction with her caregiving responsibilities. The intimate and nurturing moments shared during breastfeeding can forge enduring positive memories, contributing to a mother's happiness [17]. The tender bonds formed in these moments nurture the child and serve as sources of profound joy and contentment for the mother, enriching her emotional experience and overall well-being.

Promotion of Body Positivity and Accepting Postpartum

Breastfeeding catalyzes fostering body positivity and self-acceptance within the postpartum phase. This transformative experience empowers women to embrace the remarkable capacity of their bodies to nourish and support their newborns, leading to the cultivation of a newfound admiration for their physical forms. Breastfeeding establishes a palpable and intimate link between a mother's body and the thriving of her child, redirecting attention from societal ideals of beauty towards the empowerment found in nurturing and sustaining life. This shift in perspective can positively influence body image perceptions and bolster self-assurance amid the significant physical transformations accompanying motherhood. Mothers can find a path toward appreciating their bodies for their functional and life-sustaining role by prioritizing nurturing and nourishing through breastfeeding. This shift aligns with a broader movement of celebrating diverse body shapes and sizes, and it can contribute to an enhanced sense of self-esteem and body positivity during a period of remarkable bodily change [18].

Peer Support and Sense of Community Among Breastfeeding Mothers

Breastfeeding frequently catalyzes the formation of nurturing communities among mothers who share similar journeys. Through peer support groups, virtual discussion forums, and physical gatherings, breastfeeding mothers find a platform to connect, exchange insights, and narrate their narratives. These exchanges culminate in cultivating camaraderie and a profound sense of inclusion, addressing potential sentiments of seclusion that can manifest in new mothers. The common threads of shared experiences, encompassing challenges and triumphs, knit together a robust community fabric. This communal tapestry offers a sanctuary where mothers can gain understanding, empathy, and encouragement from those who comprehend the nuances of breastfeeding. By engaging in these networks, mothers collectively navigate the intricacies of motherhood, providing a valuable lifeline of support that bolsters their emotional and mental well-being [19]. These peer connections provide a platform for practical advice and forge a deeper connection that extends beyond breastfeeding. The shared experiences create bonds that validate the emotions and struggles that come with motherhood, ultimately contributing to an enhanced sense of belonging and improved psychological resilience. The network's ability to foster mutual understanding and shared growth significantly enhances the holistic well-being of breastfeeding mothers.

Challenges and strategies

Addressing Common Breastfeeding Difficulties and Their Impact

Breastfeeding can come with various challenges, including sore nipples, engorgement, latch problems, and low milk supply. These challenges, if not adequately addressed, can significantly impact a mother's breastfeeding experience and overall well-being. Persistent difficulties can lead to frustration, stress, and a sense of failure. It is important to acknowledge these challenges, seek timely support from healthcare professionals or lactation consultants, and explore strategies to overcome them. Addressing breastfeeding difficulties effectively can help prevent adverse psychological outcomes and foster a more positive experience for both mother and child [20].

Importance of Education and Lactation Support for Successful Breastfeeding

Education and access to lactation support are crucial factors in ensuring successful breastfeeding and promoting maternal psychological well-being. Providing expectant mothers with accurate information about breastfeeding during prenatal care equips them with the knowledge and confidence to navigate potential challenges. Lactation consultants and breastfeeding support groups offer practical guidance and emotional encouragement, helping mothers overcome obstacles and build positive breastfeeding relationships. By offering education and ongoing support, healthcare providers can empower mothers to make informed decisions and feel more capable in their breastfeeding journey [21].

Overcoming Societal Barriers and Promoting Breastfeeding-Friendly Environments

Societal barriers, such as cultural norms, workplace policies, and lack of public support, can hinder breastfeeding success and contribute to maternal stress. Creating breastfeeding-friendly environments is essential to alleviate these barriers. Implementing workplace policies that support breastfeeding mothers, ensuring access to private and comfortable spaces for pumping, and promoting public acceptance of breastfeeding can significantly improve the breastfeeding experience. By actively addressing societal challenges, communities can foster an environment where mothers feel empowered to breastfeed confidently and without fear of judgment [22].

Cultural and societal influences

Cultural Perspectives on Breastfeeding and Maternal Well-being

Cultural beliefs and practices play a significant role in shaping attitudes toward breastfeeding and maternal well-being. In some cultures, breastfeeding is viewed as a natural and integral aspect of motherhood, closely linked to a woman's identity and sense of purpose. In others, cultural norms and misconceptions may influence perceptions of breastfeeding, affecting a mother's confidence and self-esteem. Recognizing and respecting diverse cultural perspectives is essential for promoting positive maternal well-being. Educating communities about the psychological benefits of breastfeeding while acknowledging cultural values can contribute to a more inclusive and supportive environment for mothers [23].

Shifting Societal Attitude Towards Breastfeeding in Public

Societal attitudes toward breastfeeding in public spaces have evolved, with advocacy efforts aiming to normalize this natural behavior. Despite progress, lingering discomfort or stigma associated with breastfeeding in public can contribute to a mother's stress and anxiety. Shifting societal attitudes requires public education campaigns that emphasize the importance of breastfeeding and create awareness about the rights of breastfeeding mothers. Encouraging open conversations and fostering an atmosphere of acceptance can alleviate mothers' concerns and contribute to their overall well-being [24].

Policy Implications for Supporting Breastfeeding Mothers

Government policies and institutional support play a vital role in supporting breastfeeding mothers. Policies that mandate reasonable accommodations for breastfeeding mothers in the workplace, such as designated pumping spaces and flexible break times, can alleviate stress and enable mothers to continue breastfeeding while returning to work. Additionally, policies that support maternity leave and access to lactation consultants ensure that mothers have the time and resources they need to establish successful breastfeeding practices. Collaborative efforts between governments, healthcare institutions, and workplaces are essential for creating an environment prioritizing maternal well-being through breastfeeding-friendly policies [25].

Weaning and transition

Emotional Worlds of Weaning for Both Mother and Child

Weaning, transitioning a baby from breastfeeding to other forms of nourishment, is an emotionally significant phase for both mother and child. For mothers, weaning can evoke emotions ranging from pride and accomplishment to sadness and nostalgia as a cherished chapter ends. Similarly, children may experience various emotions as the familiar source of comfort and nourishment changes. Understanding and acknowledging these emotions is crucial in ensuring both parties' supportive and empathetic transition [26].

Strategies for Facilitating a Smooth Transition From Breastfeeding

To facilitate a smooth transition from breastfeeding, several strategies can be employed. Gradual weaning, wherein breastfeeding sessions are gradually reduced over time, can help the mother and child adjust to the change. Introducing alternative feeding methods, such as bottle-feeding or introducing solid foods, can help the child adapt to new ways of receiving nourishment. Creating consistent routines and maintaining nurturing interactions, even without breastfeeding, can provide a sense of continuity and security for the child. Clear communication and transparency with the child, especially for older infants and toddlers, can help them understand the process and cope with the changes [27].

Maintaining Emotional Connection Beyond Breastfeeding

While breastfeeding may transition, the emotional connection between mother and child remains vital. Engaging in skin-to-skin contact, cuddling, and maintaining eye contact during feedings can continue to provide emotional intimacy and reassurance for both parties. Finding alternative bonding activities, such as reading, playing, and spending quality time together, can help strengthen the emotional bond beyond breastfeeding. The emotional connection formed during breastfeeding can extend and evolve into other forms of closeness and attachment as the child grows [13].

## Conclusions

The psychological advantages of breastfeeding are extensive and profound. The interaction of hormones, facilitated by oxytocin and prolactin, fosters a strong maternal bond, reduces the risk of postpartum depression and anxiety, and enhances self-confidence. Additionally, breastfeeding simultaneously facilitates optimal brain development, a robust immune system, and cognitive-emotional growth in children through maternal interaction. This mutual relationship fosters secure attachment and emotional well-being. Addressing this matter requires immediate action to support breastfeeding and enhance awareness. This involves prioritizing lactation education, offering accessible assistance, and cultivating environments that normalize breastfeeding as a cherished practice. Elevating public awareness about the comprehensive benefits of breastfeeding helps dispel misconceptions, eliminate stigma, and empower mothers to make informed choices in nurturing their infants. The far-reaching implications of breastfeeding involve maternal well-being, child development, and society. They fortify maternal mental health and confidence, contributing to healthier family dynamics. The cognitive, emotional, and immunological advantages gained by children establish a promising foundation for their future. On a societal level, endorsing and fostering breastfeeding create a nurturing environment that values the health and happiness of both mothers and their children. Breastfeeding represents a transformative journey that encompasses more than just nutrition - it represents a complex array of interconnected benefits that resonate throughout our lives. By championing awareness and support for breastfeeding, we contribute to a world that prioritizes the comprehensive health of mothers and their children, fostering thriving relationships.
